# P110: Influence of signal coloured hand disinfectant dispensers on compliance at an intensive care unit

**DOI:** 10.1186/2047-2994-2-S1-P110

**Published:** 2013-06-20

**Authors:** S Scheithauer, H Haefner, J Schroeder, K Lewalter, V Krizanovic, S Lemmen

**Affiliations:** 1 University Hospital Aachen, RWTH Aachen, Aachen, Germany

## Introduction

Improvement of hand hygiene compliance in all-day setting is crucial and thus a multifaceted approach is generally advocated.

## Objectives

In order to assess the influence of signal coloured hand disinfectant dispensers the number of dispenser activities was documented and analyzed in comparison to historical data and with respect to the number of health-care workers.

## Methods

In a 14-bed cardiology ICU all 25 standard dispensers were exchanged with signal-coloured dispensers (Ophardt Hygiene-Technik; Issum, Germany). Dispenser activities were documented dispenser-specifically for 12 weeks and analyzed by week. The use of gown pocket dispensers was forbidden during the investigation. Health care workers had been trained in hand hygiene for the last three years continuously.

## Results

A total of 81654 dispensers activities translating to 40827 hand rubs were documented with the majority (72%) occurring at patient-near dispensers (58852 activities, 29426 hand rubs). There was no time-dependency with dispenser activities ranging from 6119 to 8026 per week. Taking the number of patient days into consideration these activities led to 39 to 41 hand-rubs per patient day. This ranges between the 75. and 90. percentile in the national benchmark. Comparison with historical data revealed no difference. However, the nurse to patient ratio decreased during the study period by 8.5%. Taking an increased workload into consideration (hand rubs divided by patient-days and number of health-care workers) revealed a 6% increase in staff-adjusted compliance.

## Conclusion

Signal-coloured dispensers seem to be a useful additional tool to improve and maintain hand hygiene compliance even in ICUs with an already good hand hygiene compliance.

## Disclosure of interest

None declared

